# Items

**Published:** 1878-10

**Authors:** 


### Items.

INDIANA, ILLINOIS AND KENTUCKY TRI-STATE MEDICAL SOCIETY.—The fourth annual meeting of this society, will be held in the city of Springfield, Ills., November 13, 14 and 15, 1878, commencing at 11 a. m., 13th.

Extract from plan of organization.—"All members in good "standing of any local, county, district or State medical societies, "auxiliary to the American Medical Association, may become "members on payment of the fees and a vote of the society.

G. W. BURTON, M. D., Secretary, Mitchell, Ind.

B. M. GRIFFITH, M. D.,
Chairman Com. Arrangements, Springfield, Ills.

THE "ANNUAL CIRCULAR AND CATALOGUE OF THE COLLEGE OF PHYSICIANS AND SURGEONS, city of Keokuk, Iowa," an affair of eight pages, is again before us. From it we learn that "J. C. Hughes, M. D.," is President of the Board of Curators, Professor of the Institutes and Practice of Surgery and Surgical Clinics (sic), Dean of the Faculty, and also Surgeon-in-charge of the Medical and Surgical Infirmary and Eye and Ear Institute. "J. C. Hughes, Jr., M. D.," is Professor of Anatomy, and, also, as appears from an advertisement on the eighth and last page of the "Circular and Catalogue," has "a large and complete assortment of surgical instruments. Orders promptly attented (sic) to." Four other gentlemen make up the list of professors, two of them, we are told, are now in Europe, but will return "ripe with experience and ready to impart all the old, and everything new which their observation may add as an improvement in the departments over which they preside."

We make the following excerpta from this "Circular and Catalogue" without comment: "Years of experience has (sic) proved the great advantage of extemporaneous lectures." . . . "Board can be obtained in the city at from $2.00 to $4.00 per week, *owing to the accommodations!*" "Text-books: *Cruvilhier* (!), *Aitkin* (!); *Erichson* (!), *Rockatansky* (!), *Cazeau* (!)."

The announcement of the spring session for 1879 lacks its verb. In the name of Hippocrates and Galen, let us pray that it will lack nothing more! Fees for the Course, $20.00!

Locust Oil.—Analysis and examination of the dead Rocky Mountain locusts by the United States Entomological Commission show that these insects furnish a new oil which will be christened *caloptine*, and a very large percentage of pure formic acid. Though this acid exists in the ant and some other insects, it is with difficulty obtained in large quantities; whereas by the action of sulphuric acid upon the locust juices, it passes off with great readiness, and in remarkable quantity and gravity. The various uses of this acid as a therapeutic, etc., are capable of great and valuable extension, where it can be obtained so readily, and in such quantity.—*Druggists' Circular.*

The introductory lectures to the regular winter courses of the medical schools in this city are announced as follows: For the Chicago Medical College, by Dr. Wm. E. Quine, on the evening of the 1st inst.; for the Woman's Hospital Medical College, by Dr. W. Godfrey Dyas, on the evening of the 2d inst.; for Rush Medical College, by Dr. James Nevins Hyde, on the evening of the 1st inst.

We have received the printed inaugural dissertations of two Milwaukee physicians, presented for the degree of the doctorate in the Ludwig-Maximilian University, of Munich; one, that of our correspondent, Dr. Nicholas Senn, on the subject of Varicocele; the other, that of Dr. Alois Græetinger, on Placental Arrest after Labor at Term.

